# Arabic Translation, Cultural Adaptation, and Validation of the Hyperhidrosis Disease Severity Scale (Ar-HDSS)

**DOI:** 10.3390/healthcare13040397

**Published:** 2025-02-12

**Authors:** Nasser M. AbuDujain, Qais A. Almuhaideb, Khalid M. Alghamdi

**Affiliations:** 1 University Family Medicine Center, King Saud University Medical City, King Saud University, Riyadh 11481, Saudi Arabia; nasserabudujain@gmail.com; 2 Department of Dermatology, King Faisal Specialist Hospital & Research Centre, Riyadh 11211, Saudi Arabia; qaissalmuhaideb@gmail.com; 3 Department of Dermatology, College of Medicine, King Saud University, Riyadh 11472, Saudi Arabia; 4 Vitiligo Research Chair, College of Medicine, King Saud University, Riyadh 11472, Saudi Arabia

**Keywords:** hyperhidrosis, HDSS, validity, reliability, Arabic, Saudi Arabia

## Abstract

**Background:** Hyperhidrosis (HH) is characterized by excessive sweating, which affects quality of life. The Hyperhidrosis Disease Severity Scale (HDSS) is a four-point scale used to evaluate HH severity by measuring how much excessive sweating disrupts daily activities. This study aimed to translate, validate, and adapt the HDSS tool into Arabic. **Methods:** A quantitative, analytical, cross-sectional study was carried out from May to June 2024 on patients clinically diagnosed with HH. The process of translating the HDSS into Arabic involved three independent forward translations, followed by a preliminary version created by a reviewer. Three additional independent translators conducted backward translations. All of the versions were then revised and merged to produce the final version. Reliability was evaluated through a test–retest reliability approach to ensure the reproducibility of the results. For validity, we used construct validity to compare the HDSS with the HidroQoL index. **Results:** A total of 167 patients were included, with a mean age of 29 ± 9.02 years, and over half of the patients were male (61%). The interrater agreement between the HDSS test and the retest results was substantial, with a kappa coefficient of 0.732. Significant positive correlations were observed between the HDSS score and daily life (r = 0.413, *p* < 0.001), the psychological domain (r = 0.374, *p* < 0.001), and HidroQOL (r = 0.425, *p* < 0.001). **Conclusions:** Our findings demonstrate that the Arabic HDSS has excellent psychometric properties, including construct validity and reproducibility. Proper use of the Arabic HDSS will allow the effective assessment of HH severity.

## 1. Introduction

Hyperhidrosis (HH) is a disorder characterized by increased sweating due to overstimulation of cholinergic receptors on eccrine glands, which may significantly affect quality of life [1,2]. It is classified as primary or secondary, where the primary disease typically presents earlier in life with more localized symptoms. In contrast, secondary disease typically presents as an adverse effect of medications or systemic disorders, particularly neurologic disorders [2].

Disproportionate perspiration is sometimes the cause of significant emotional distress in patients, and patients are sometimes reluctant to inform physicians of their symptoms. For this reason, researchers emphasize that the prevalence of HH is highly underreported [3]. Various epidemiological studies have been conducted to estimate the prevalence of primary HH [3,4,5,6,7,8,9,10]. Large studies conducted in the United States on samples from the general population have reported a prevalence ranging from 2.9% to 4.8% [6,7]. In European population, studies have shown a prevalence of 16.3% in Germany (employees from approximately 50 companies) and 8% in Poland (medical and dentistry students) [3,8]. In Asian populations, the prevalence of HH was estimated to be 13.95% in Japan (employees/students from 20 companies or schools) and 14.5% in China (patients from one hospital) [9,10]. In Arabian populations, two studies assessed the prevalence of primary HH; one study was performed in five hospitals in Jordan and included 4500 outpatient clinic attendants, and the other was conducted with residents in Al-Ahsa city in Saudi Arabia. The prevalence rates of primary HH in these studies were 3.2% and 18.3%, respectively [4,5].

There are several treatment options for HH. Topical aluminum chloride and oral anticholinergic medications are usually sufficient for treating mild to moderate disease [1]. Other treatments include iodophoresis, which is effective for palmar and plantar HH; botulinum toxin injections, which are safe and effective for focal HH; and energy-delivering devices [2]. Energy-delivering devices include lasers, ultrasound technology, microwave thermolysis, and fractional microneedle radiofrequency [2]. Surgery, such as excision, curettage, liposuction, or a combination of these techniques, may be considered when more conservative treatments have failed. Another surgical treatment is sympathectomy, which is considered a last-resort treatment when conservative treatments are unsuccessful or intolerable [2].

The Hyperhidrosis Disease Severity Scale (HDSS) is a four-point scale used to assess the severity of HH on the basis of the extent of excessive sweating-related impairment of daily activities [11]. It is commonly used in routine clinical practice or clinical research and can be administered by an interviewer or self-completed by the patient [11]. The HDSS has been translated and validated in Portuguese and shown to be reproducible in a Brazilian sample [12]. Despite its global acceptance and use in the Arabian population [4,5], the HDSS has not been validated or professionally translated into Arabic, limiting its use in the Arabian population. Consequently, this project aimed to translate and validate the HDSS into Arabic.

## 2. Methodology

### 2.1. Study Design, Participants, and Setting

A quantitative, cross-sectional validation study was conducted in the Dermatology clinics at King Saud University Medical Center in Riyadh, Saudi Arabia, from May 2024 to June 2024. A total of 167 participants were recruited, all fluent Arabic speakers aged 15 years or older and diagnosed with primary hyperhidrosis. Non-native Arabic speakers, those younger than 15 years of age, and individuals with hyperhidrosis due to a secondary treatable cause were excluded. The sample’s demographic characteristics highlight the diversity of the study population in terms of age, gender, education, and income.

### 2.2. Questionnaire

The participants completed an electronic survey comprising three sections: (1) demographic information, (2) medical history related to HH, and (3) the Hyperhidrosis Disease Severity Scale (HDSS) tool and Hyperhidrosis Quality of Life (HidroQol) Index. The demographic section collected data on age, sex, level of education, and monthly income. The medical history section involved the age of diagnosis with HH, affected sites, therapeutic interventions used, and past medical history of chronic medical illness. The last section includes the Arabic-translated version of the HDSS, along with the HidroQol©.

#### 2.2.1. Hyperhidrosis Disease Severity Scale

The HDSS is a self-administered tool developed by Kowalski and colleagues in 2004 [11]. It is intended to measure the severity of hyperhidrosis experienced by a patient with only one item: “How would you rate the severity of your hyperhidrosis?” The answers to the question consist of four choices: 1 = “My sweating is never noticeable and never interferes with my daily activities”, 2 = “My sweating is tolerable but sometimes interferes with my daily activities”, 3 = “My sweating is barely tolerable and frequently interferes with my daily activities”, and 4 = “My sweating is intolerable and always interferes with my daily activities”. A score of 3 or 4 indicates severe hyperhidrosis. A score of 1 or 2 indicates mild or moderate hyperhidrosis. Some experts tailor management plans on the basis of the score. The HDSS has excellent psychometric properties, including test–retest reliability (r = 0.82; *p* < 0.05) and construct validity (r = 0.35–0.77; *p* < 0.001).

#### 2.2.2. Hyperhidrosis Quality of Life Index

The HidroQoL© is another self-administered, patient-reported outcome tool developed by P. Kamudoni in 2015 [13]. It works by assessing the quality of life among those affected by HH via several Likert-scale questions. It consists of 18 items that fall under two main domains. Domain 1 assesses activities of daily life, and Domain 2 assesses psychosocial life. Each item contains three choices (very much [0 points], a little [1 point], and no, not at all [2 points]). The final score can range between 0 and 36, which is calculated by summing the scores. If the total score was = 0–1, it indicates that the disease has no effect at all; if = 2–11, it indicates that the disease has a small effect; if = 12–22, it indicates that the disease has a moderate effect; if = 23–32, it indicates that the disease has a large effect; and if 33–36, it indicates that the disease has a very large effect. The HidroQoL© has undergone further validation in recent years by Donhauser et al. [14] and Gabes et al. [15] from Germany, who reported excellent internal consistency and strong test–retest reliability (Cronbach’s α 0·81–0·90; ICCs 0·89–0·93) along with satisfactory construct validity. We used the Arabic version of the HidroQoL©, which underwent psychometric validation by Almuhaideb et al. in 2025 and has good psychometric properties (in press) [16].

### 2.3. Instrument Translation Process

A robust translation process was employed, beginning with obtaining permission from the publisher. Three independent professional translators performed forward translations, creating the TL-1, TL-2, and TL-3 versions. Another translator who was not previously familiar with the tool for producing a preliminary version (PI-TL) compared and contrasted the three versions. Another three independent professional translators performed the backward translations, creating B-TL 1, B-TL 2, and B-TL 3. All three versions were subsequently revised and merged, producing the final version (FTL). The rationale for employing this rigorous translation process was to address potential cultural and linguistic differences that might affect the interpretation of the tool. Forward translation ensures semantic accuracy, while backward translation helps detect discrepancies and verifies that the translated version faithfully represents the original tool’s intent. This dual-step process is essential for developing psychometrically valid instruments that are culturally appropriate for the target population. A Supplementary File containing the Arabic version of HDSS (Ar-HDSS) is attached [Supplementary File S1].

### 2.4. Psychometric Analysis for Validity and Reliability

To assess the reliability of the Ar-HDSS, we conducted test–retest reliability tests to assess the stability of the items over time. The test–retest reliability assessment was conducted by administering the Ar-HDSS to the same participants twice, with a one-week interval between the tests. Internal consistency was not assessed, as it is statistically unfeasible to calculate Cronbach’s alpha for one item, as in the HDSS. To assess the reliability of the Ar-HDSS, we used construct (convergent) validity to correlate the Ar-HDSS and the Ar-Hidro-Qol.

### 2.5. Ethical Considerations

Ethical approval was obtained from the Institutional Review Board at the College of Medicine, King Saud University, in February 2024. The participants provided electronic consent after being informed of the study’s purpose, expected completion time, principal investigator’s contact information, and their right to withdraw without obligation. Anonymity was ensured by not collecting identifying data, and no incentives were provided.

### 2.6. Statistical Analysis

The data were analyzed via SPSS (Statistical Package for the Social Sciences) version 27. Descriptive statistics were used to calculate the numbers and percentages for qualitative variables. Mean, standard deviation, and range are presented for quantitative variables. Reliability was evaluated via the test–retest reliability of the Ar-HDDS total score, which was determined by the intraclass correlation coefficient (ICC). Spearman correlations were tested between HidroQOL scores and other variables to assess construct validity. The Bland–Altman graph was used; this is a valuable tool for assessing the agreement between two different measurement methods. This graphical method plots the differences between the two methods against their average, allowing the clear visualization of any systematic bias or discrepancies.

## 3. Results

A total of 167 participants were enrolled in this study; more than one-half were males (61.1%). The age range of the participants was 16–63 years, with a mean ± SD of 29 ± 9.02 years. The large majority of the participants were of Saudi descent (one-hundred fifty-five (92.8%) participants), and most of the participants had a bachelor’s degree (75.4%). The largest proportion reported a monthly income of less than SAR 5000 (44.9%). Only twenty-six (15.7%) reported having a chronic illness (Table 1). The patients’ medical histories are shown in Table 2. The locations most affected by HH were the palms (79.6%) and soles of the feet (74.9%). In terms of the number of sites reported, the median score was two, and the most reported number of sites was two (40.7%). The majority of HH was managed by topical antiperspirants (46.7%), whereas the least common management method was oral antiperspirants (5.4%). Thirty-one (18.6%) reported the use of no treatment.

**Figure 1:**
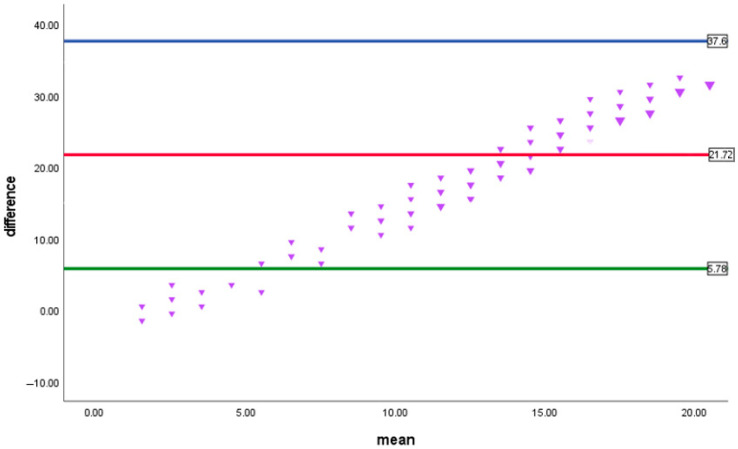
Bland–Altman plot between the HDSS score and HidroQoL showing a consistent positive bias (~21.72) between the HDSS score and HidroQoL, with potential proportional bias as differences increase with higher mean values.

The severity of hyperhidrosis in patients is shown in Table 3. The largest proportion, eighty-nine (53.3%), reported that their sweating is intolerable and always interferes with their daily activity, whereas the smallest proportion (1.8%) reported that their sweating is never noticeable and never interferes with their daily activity. Table 4 reveals the correlation between the HDSS score and quality of life. Significant positive correlations were found between the HDSS score and daily life (r = 0.413, *p* < 0.001), psychological domain (r = 0.374, *p* < 0.001), and HidroQOL (R = 0.425, *p* < 0.001) (Figure 1). On the other hand, there was a significant negative correlation between the HDSS score and age at HH onset (r = −0.231, *p* = 0.033). No correlations were found between the HDSS score and the site-of-involvement score or age.

The correlation between the test and retest results showed no significant variations (*p* = 0.7), where the mean ± SD of the HDSS score in the test was 3.26 ± 7.52, and the mean ± SD of the HDSS score in the retest was 3.13 ± 0.815, with a mean difference ± SD of 0.13 ± 0.55 (Table 5). The interrater agreement between the HDSS test and the retest was substantial, and the kappa coefficient was 0.732 (Table 5).

## 4. Discussion

Hyperhidrosis is a medical condition that can have many debilitating effects on affected patients, including personal, psychosocial, and sexual effects. This study aimed primarily to translate the HDSS tool into Arabic to better understand HH severity in Arab communities and allow better local research on the topic. Our study confirms that the one-item Arabic HDSS is valid and reliable for use in assessing the severity of HH among affected individuals.

Our study confirms that the one-item Arabic HDSS is valid and reliable for use in assessing the severity of HH among affected individuals. Importantly, the process of cultural adaptation went beyond linguistic translation to ensure the tool’s relevance and clarity for Arabic-speaking populations. This adaptation accounted for cultural norms, health beliefs, and idiomatic expressions. For example, we paid special attention to how terms related to sweating and its impact on daily life were phrased to align with the cultural sensitivity and understanding of hyperhidrosis within the Arab community. Additionally, cultural factors that influence how individuals perceive and report health conditions, particularly those related to personal hygiene, were incorporated to make the tool practical and meaningful in this context. This comprehensive approach was critical for ensuring that the Ar-HDSS maintains the validity and reliability of the original tool while being appropriate for the target population.

The inclusion of demographic variables such as education level and monthly income provides valuable context for understanding hyperhidrosis in the studied population. These factors may significantly influence awareness, treatment-seeking behaviors, and access to resources. For instance, higher education levels may correlate with better understanding of hyperhidrosis and more proactive management, while income levels can impact the affordability and accessibility of advanced treatments. Although not the primary focus of this study, analyzing these demographic characteristics offers insights into how socio-economic factors might shape the experiences and management of hyperhidrosis.

To assess the reliability of the Ar-HDSS, we only employed the test–retest method to assess stability and agreement, as internal consistency is statistically not applicable for a one-item scale [17]. Our analysis revealed good test–retest reliability and agreement, with values of 0.76 and 0.73, respectively. This is comparable to the original HDSS validation study conducted by Kowalski et al. [11], where the test–retest value was 0.82; however, this value is higher than that reported in the Portuguese validation study [12]. For the validity of the Ar-HDSS, we assessed the construct (convergent) validity by assessing the correlation with the Arabic Hyperhidrosis Quality of Life Index (HidroQOL©). While the original study by Kowalski et al. employed the Dermatology Life Quality Index (DLQI) and the Hyperhidrosis Impact Questionnaire (HHIQ), the DLQI was validated in Arabic only among psoriasis patients [18], which restricts its usability for other conditions, such as HH, and the HHIQ has not yet been validated and culturally adapted into Arabic. A significant strong positive correlation was found between the HDSS score and the HidroQOL© score (an overall positive correlation, as well as a positive correlation with its subdomains), with a Spearman’s rho of 0.43 for the overall tool, and 0.41 and 0.37 for the subdomains (1) Daily life and (2) Psychological, respectively. The Ar-HDSS score was negatively correlated with age at HH onset, which makes sense, as individuals become accustomed to their underlying condition as they grow older and experience this condition for longer durations.

The collected sample size is adequate and supports the statistical analyses and positive findings of this study. However, certain limitations must be acknowledged. First, the sample population primarily consisted of Saudi citizens, which may restrict the generalizability of the findings to other Arabic-speaking populations. Future studies should include participants from diverse regions to ensure broader applicability. Second, validation was performed using a single instrument, the HidroQoL, as a gold-standard measure for hyperhidrosis severity is not currently available. Including additional validation tools or new standardized measures in future research could strengthen the findings. Lastly, this study did not evaluate the Ar-HDSS for sensitivity to clinical changes or its applicability in longitudinal settings, which limits its use for tracking treatment responses over time. Future research should address these gaps by exploring the tool’s responsiveness to interventions, its predictive validity, and its applicability in various clinical settings, such as primary care and dermatology clinics.

## 5. Conclusions

Our study demonstrated that the Arabic HDSS that we produced is valid and reliable for use among Arabic-speaking individuals with HH. Effective utilization of the Ar-HDSS will reveal the severity of HH, allowing a better understanding of the condition and allowing for the development of management plans. Future studies addressing HH in the region are warranted to allow for a better understanding of such a condition.

## Figures and Tables

**Table 1:** Demographic characteristics of 167 subjects.

Variable		n (%)
Sex	Male	102 (61.1%)
	Female	65 (38.9%)
Age	Mean ± SD	29 ± 9.02
	Range	16–63
Nationality	Saudi	155 (92.8%)
	Non-Saudi	12 (7.2%)
Level of education	High-school and below	27 (16.2%)
	Bachelor’s degree	126 (75.4%)
	Postgraduate degree	14 (8.4%)
Monthly income	Less than SAR 5000	75 (44.9%)
	from SAR 5000 to less than SAR 15,000	59 (35.3%)
	from SAR 15,000 to less than SAR 30,000	29 (17.4%)
	More than SAR 30,000	4 (2.4%)
Chronic medical illness	Yes	26 (15.7%)
	No	141 (84.3%)

**Table 2:** Medical history.

Affected Regions	n (%)
Axilla	97 (58.1%)
Palms	133 (79.6%)
Soles of the feet	125 (74.9%)
Body trunk	34 (20.4%)
Face and scalp	41 (24.6%)
Genitalia	25 (15.0%)
**Scores computed for the number of sites affected**	
One site only	20 (12.0%)
Two sites	68 (40.7%)
Three sites	42 (25.1%)
Four sites	21 (12.6%)
Five sites	8 (4.8%)
Six sites	8 (4.8%)
Median (IQR)	2 (2–3)
**Management used for HH**	
None	31 (18.6%)
Topical antiperspirants (such as aluminum chloride)	78 (46.7%)
Oral antiperspirants (such as glycopyrrolate or oxybutynin)	9 (5.4%)
Botulinum toxin injection	56 (33.5%)
Anti-hyperhidrosis machine (iontophoresis, radiofrequency, etc.)	16 (9.6%)
Surgical intervention (such as thoracic sympathectomy)	72 (43.1%)

**Table 3:** “How would you rate the severity of your hyperhidrosis?”.

Statement	n (%)
My sweating is never noticeable and never interferes with my daily activities.	3 (1.8%)
My sweating is tolerable but sometimes interferes with my daily activities.	31 (18.6%)
My sweating is barely tolerable and frequently interferes with my daily activities.	44 (26.3%)
My sweating is intolerable and always interferes with my daily activities.	89 (53.3%)

**Table 4:** Correlation between HDSS score and quality of life questionnaire and other factors.

	r	*p* Value
Daily life domain	0.41 *	<0.001
Psychological domain	0.37 *	<0.001
HidroQoL	0.43 *	<0.001
Sites of involvement score	0.08	0.33
Age	0.1	0.2
Age since HH started	−0.23	0.03

* Spearman’s rho.

**Table 5:** HDSS score test–retest correlation and agreement.

Test–Retest Correlation					
		**Mean**	**Std. Deviation**	**Mean Difference**	R^2^ **(*p* Value)**
HDSS tool	(test)	3.26	0.75	0.13 ± 0.55	0.76
	(retest)	3.13	0.81		
**Test–retest level of agreement**					
		HDSS tool (retest)			Kappa measure of agreement
		2	3	4	
HDSS tool (test)	2	4	0	0	0.73
	3	1	7	1	
	4	1	1	8	

## Data Availability

Data used in this study are available upon reasonable request from the corresponding author.

## Supplementary Materials

The following supporting information can be downloaded at: https://www.mdpi.com/article/10.3390/healthcare13040397/s1.
